# A Twist in Crohn’s Disease: A Rare Case of Gastric Volvulus

**DOI:** 10.7759/cureus.10158

**Published:** 2020-08-31

**Authors:** Zain U Abideen, Rehman M Bhatti, Ali Jaan, Zahoor Ahmed, Pervaiz Laghari

**Affiliations:** 1 Internal Medicine, Abington Hospital–Jefferson Health, Abington, USA; 2 Internal Medicine, Ayub Medical College, Abbottabad, PAK; 3 Internal Medicine, King Edward Medical University–Mayo Hospital, Lahore, PAK; 4 Medicine, New Medical Center (NMC) Specialty Hospital, Abu Dhabi, ARE

**Keywords:** crohn's disease, stricture, gastric volvulus

## Abstract

An 80-year-old male with a past medical history of Crohn's disease presented to the emergency department with complaints of nausea and multiple episodes of coffee-ground emesis and was initially diagnosed with upper gastrointestinal bleed. On physical examination, the patient was noted to have a mildly tense and tender abdomen with hyperactive bowel sound. His CT abdomen showed a markedly distended stomach with mesenteroaxial (MA) rotation and localized fluid in the left upper quadrant with the caudal displacement of the spleen due to the left upward position of the stomach. The gastric outflow tract was pinched to the left of the midline superior to the esophagogastric junction, consistent with the gastric volvulus. Endoscopic detorsion was initially planned, but it was unsuccessful due to the twisting of the distal stomach in the antrum. The patient underwent laparoscopic detorsion and gastropexy. He was found to be asymptomatic after the procedure and was discharged with outpatient follow-up. Gastric volvulus is a rare yet potentially fatal condition due to its variable presentation, and it can have lethal consequences if not treated properly and in a timely manner.

## Introduction

Gastric volvulus is not a commonly encountered medical condition; hence, even though it is a potentially treatable disease, its diagnosis can often be missed. Clinicians should maintain a high index of suspicion for an appropriate and timely diagnosis of the condition. If not diagnosed and treated early, gastric volvulus can lead to severe complications like gastric ischemia with necrosis, gastric perforation, pancreatic inflammation, and splenic rupture. The mortality rate of gastric volvulus is as high as 60%, making this condition potentially fatal and necessitating a rapid diagnosis followed by appropriate intervention, to avoid the complications [1]. In this report, we present a rare case of an elderly male, with a longstanding history of Crohn's disease and chronic gastric distension, who presented with gastric volvulus.

## Case presentation

An 80-year-old male with a past medical history of Crohn's disease presented to the emergency department with complaints of nausea and multiple episodes of coffee-ground emesis for one day and was initially diagnosed with upper gastrointestinal bleed. His prior medical conditions included longstanding Crohn's disease, hypertension, diabetes, and coronary artery disease. He had undergone previous abdominal surgery for the complication of small intestinal obstruction due to Crohn's disease in 2017. On initial evaluation, he had a blood pressure of 159/74 mmHg, pulse of 84 per minute, respiratory rate of 18 per minute, oxygen saturation levels (SpO_2_) of 98% on room air, and body weight 125 pounds. On physical examination, the patient was noted to have a mildly tense and tender abdomen with hyperactive bowel sound. The fluid thrill and shifting dullness were negative.

**Figure 1:**
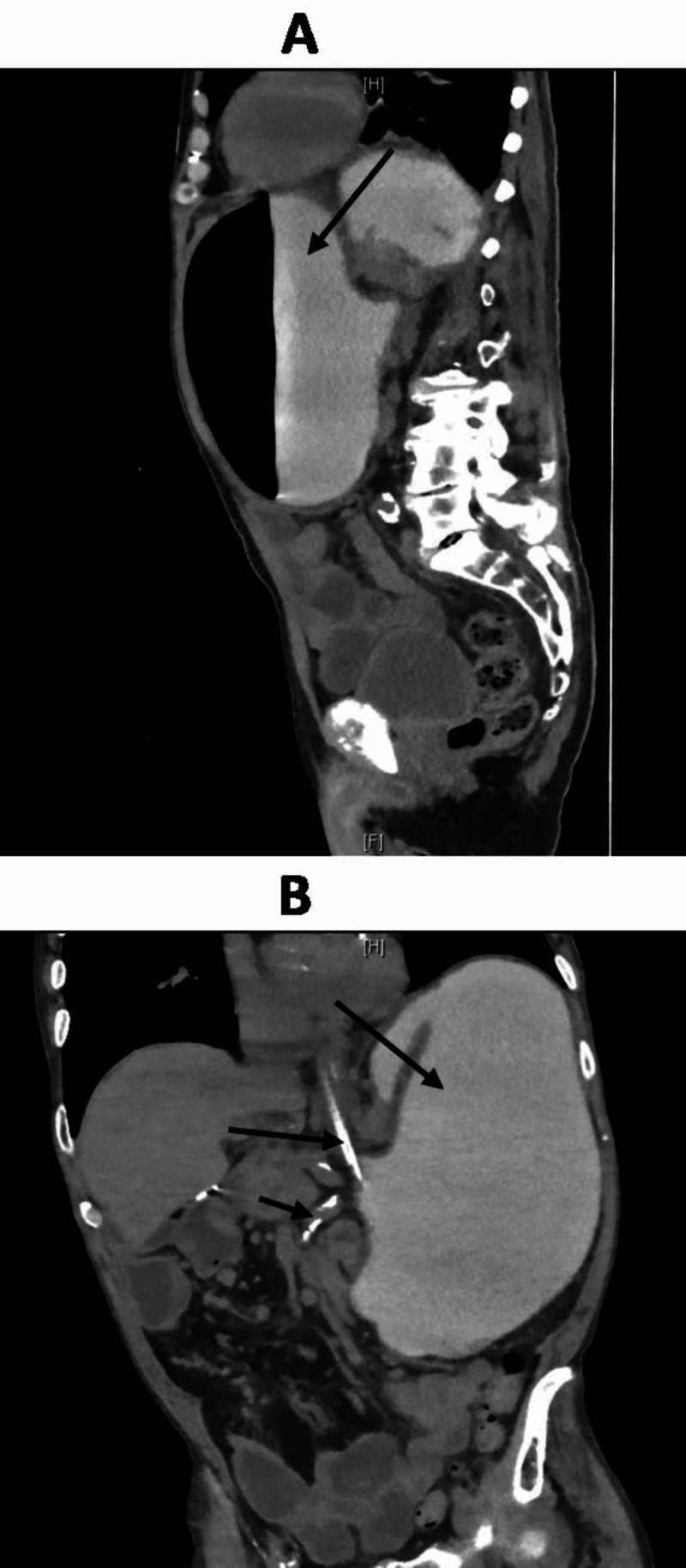
Coronal (A) and sagittal (B) views of CT abdomen with oral contrast Findings are consistent with a mesenteroaxial orientation or incomplete mesenteroaxial gastric volvulus with some of the contrast material passing into the duodenum and proximal jejunal loops. The focal short segment is seen narrowing at the level of the junction of the gastric antrum/duodenum. Contrast material is seen within the partially opacified stomach. In addition, it is important to note that the contrast material is passing into the duodenum and proximal jejunal loops consistent with partial obstruction secondary to the focal narrowing at the level of the gastric antrum/proximal duodenum CT: computed tomography

**Figure 2:**
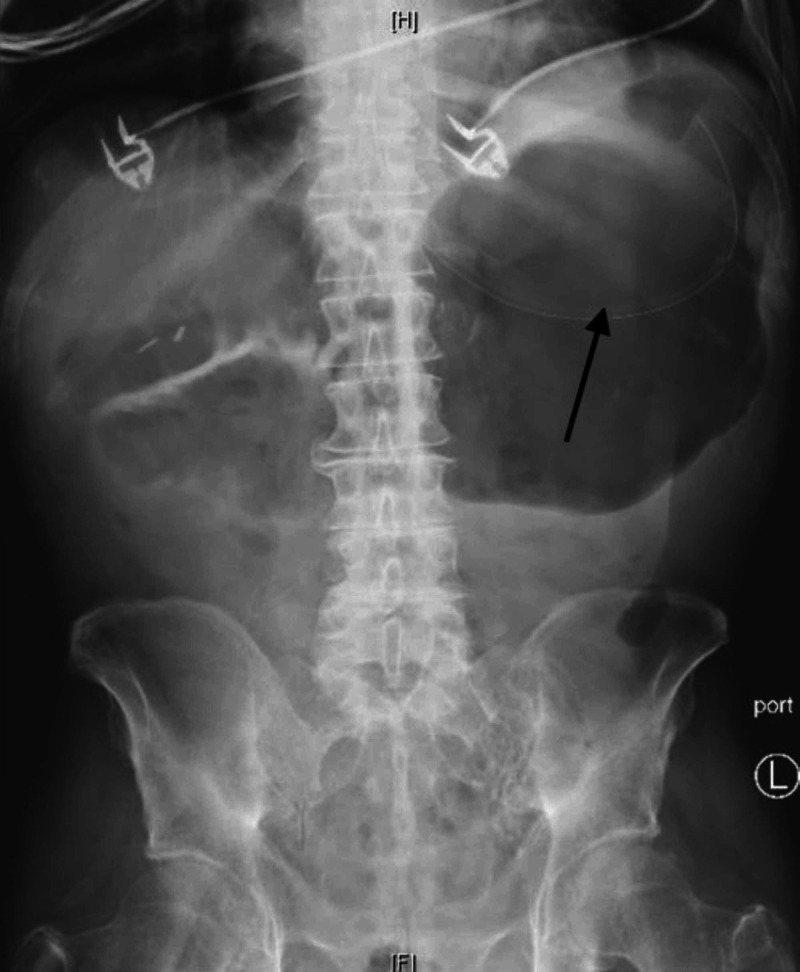
Marked gaseous distension of the stomach seen on abdominal X-ray

**Figure 3:**
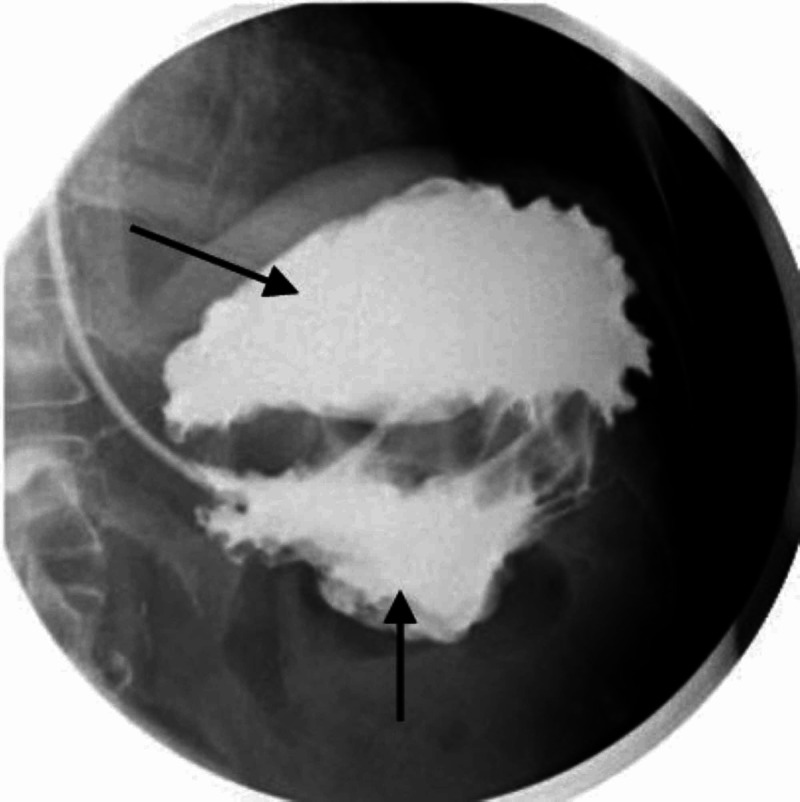
Barium swallow Findings are consistent with a mesenteroaxial orientation or incomplete mesenteroaxial gastric volvulus of the slightly decompressed stomach with some of the contrast material passing into the duodenum and proximal jejunal loops. The focal short segment is seen narrowing at the level of the junction of the gastric antrum/duodenum

His blood work revealed creatinine of 1.27 mg/dL, blood urea nitrogen of 27 mg/dL, sodium of 147 mmol/L, potassium of 3.6 mmol/L, chloride of 103 mmol/L, bicarbonate of 27 mmol/L, bilirubin of 2.3 mg/dL, lactate of 1.3 mg/dL, cardiac troponins of <0.10 (normal), WBC of 22,000 cells/mm^3^, and hemoglobin of 14.2 mg/dL. Imaging studies included CT abdomen and pelvis, which showed a markedly distended stomach filled with the enteric contrast, rotated in mesenteroaxial (MA) rotation, and localized fluid in the left upper quadrant with the caudal displacement of the spleen due to left upward stomach (Figures 1, 2). The gastric outflow tract was pinched to the left of the midline superior to the esophagogastric junction related to the gastric volvulus, with a small amount of contrast material passing into the small intestine, suggesting a short segment of focal narrowing at the level of antrum or proximal duodenum (Figures 2, 3). This was consistent with the diagnosis of gastric volvulus. After the initial resuscitation, the gastroenterologist decided to perform endoscopic detorsion, which was unsuccessful due to the twisting of the distal stomach in the antrum and stricture at the level of the antrum. The area was traversed multiple times without achieving clear untwisting due to adhesions.

The patient was taken to the operating room and laparoscopy was performed to find a redundant stomach without adhesions or masses. The stomach was detorsed. The gastroenterologist was consulted in the operating room and endoscopy was performed. The narrowing at the junction of the antrum and proximal duodenum was identified. Gastric and duodenal mucosa were healthy, without lesions or masses, and gastropexy was performed. Postoperatively, the patient was kept under observation for one week and was discharged home with outpatient follow-up. At his biweekly follow up, the patient was found to be doing well.

## Discussion

Gastric volvulus is a rare yet potentially fatal condition. It is characterized by abnormal rotation of the stomach along its axis by more than 180 degrees, leading to a variable degree of gastric inlet and outlet obstruction [2,3]. Abnormal rotation of the gut can result in gastric outlet obstruction leading to vascular ischemia, necrosis, or even perforation, making gastric volvulus a surgical emergency.

Based on etiology, gastric volvulus can be classified as primary or secondary. The primary gastric volvulus occurs due to a defect in gastric anchoring ligaments. The stomach is anchored by the gastrohepatic, gastroduodenal, gastrosplenic, and gastrocolic ligaments [4-6]. The secondary gastric volvulus is associated with a defect in the gastric anatomy as well as gastric dysfunction [1,7]. Gastric volvulus can be organoaxial (OA) and MA. In OA, the stomach rotates around an axis that connects the gastroesophageal junction and the pylorus, and in MA, the rotation usually occurs around an axis that bisects both the lesser and greater curve; it can also be of mixed type [8]. The most crucial risk factor in this type is the laxity of the gastrosplenic ligament. Crohn's disease with chronic small bowel narrowing due to strictures can lead to chronic gastric distension that causes stress on the gastric anchoring ligaments, especially the gastrosplenic ligament, which results in its laxity, leading to the risk of spontaneous gastric volvulus.

The signs and symptoms of gastric volvulus can range from incidental imaging findings to life-threatening emergencies, based on the progression of onset, volvulus type, the extent of gastric dilatation, and obstruction [9]. Symptoms can mimic those of gastritis, cholecystitis, peptic ulcer disease (PUD), or even angina pectoris [9]. A chronic gastric volvulus can present as significant chest pain with negative cardiac workup. Anemia, weight loss, dyspnea, reflux, bloating, dyspepsia, or dysphagia can be the presenting signs of chronic gastric volvulus. Patients with severe gastric volvulus may present with Borchardt's triad, which is observed in 70% of the cases.

The diagnosis of the condition is usually challenging due to its nonspecific symptoms and rarity. However, abdominal radiograph and an upper gastrointestinal series are considered the diagnostic tools of choice [10]. CT scan provides a more accurate diagnosis with particular details of the anatomical abnormalities [5,6]. Endoscopy is highly suggestive to see the distortion of any gastric anatomy. Initial management includes gastric decompression followed by surgery to check the gastric viability, reduction of the volvulus, resection of gangrenous portion, repair of the predisposing structural defects, and gastropexy to prevent a recurrence [11]. An emergent laparotomy is still a commonly used treatment option for gastric volvulus. However, laparoscopic interventions have also been described in cases of chronic gastric volvulus [12]. In our patient, healthy gastric and duodenal mucosa was identified without lesions or masses, and gastropexy was performed. The patient recovered well with no reoccurrence of symptoms.

## Conclusions

Gastric volvulus is a rare but potentially lethal condition, and it can lead to fatal consequences if not diagnosed and managed in a timely and proper manner. Due to its rarity and variable clinical presentations, a high index of clinical suspicion is required for the diagnosis of gastric volvulus. Gastric volvulus is a surgical emergency and should be considered as a differential diagnosis for any patient with a longstanding history of Crohn’s disease presenting with chronic gastric distension.
